# Mating system variation drives rapid evolution of the female transcriptome in *Drosophila pseudoobscura*

**DOI:** 10.1002/ece3.1098

**Published:** 2014-05-06

**Authors:** Elina Immonen, Rhonda R Snook, Michael G Ritchie

**Affiliations:** 1 School of Biology, University of St AndrewsDyers Brae House, St Andrews, Fife, KY16 9TH, U.K; 2 Department of Ecology and Genetics (Animal Ecology), Evolutionary Biology Centre, Uppsala UniversityNorbyvägen 18 D, Uppsala, 752 36, Sweden; 3 Animal & Plant Sciences, University of SheffieldAlfred Denny Building, Sheffield, S10 2TN, U.K

**Keywords:** *Drosophila*, gene expression, microarray, polyandry, sexual selection

## Abstract

Interactions between the sexes are believed to be a potent source of selection on sex-specific evolution. The way in which sexual interactions influence male investment is much studied, but effects on females are more poorly understood. To address this deficiency, we examined gene expression in virgin female *Drosophila pseudoobscura* following 100 generations of mating system manipulations in which we either elevated polyandry or enforced monandry. Gene expression evolution following mating system manipulation resulted in 14% of the transcriptome of virgin females being altered. Polyandrous females elevated expression of a greater number of genes normally enriched in ovaries and associated with mitosis and meiosis, which might reflect female investment into reproductive functions. Monandrous females showed a greater number of genes normally enriched for expression in somatic tissues, including the head and gut and associated with visual perception and metabolism, respectively. By comparing our data with a previous study of sex differences in gene expression in this species, we found that the majority of the genes that are differentially expressed between females of the selection treatments show female-biased expression in the wild-type population. A striking exception is genes associated with male-specific reproductive tissues (in *D. melanogaster*), which are upregulated in polyandrous females. Our results provide experimental evidence for a role of sex-specific selection arising from differing sexual interactions with males in promoting rapid evolution of the female transcriptome.

## Introduction

During sexual interactions, each sex forms the social environment against which the other sex evolves (Wolf et al. 1998). Changes in the operational sex ratio alter the sexual environment and thus the strength of sexual selection (Kokko et al. 2006). Changes in operational sex ratio also change the mating system. Mating systems are a key factor in many evolutionary processes, including sexual selection (Birkhead and Pizzari 2002), speciation (Martin and Hosken 2003; Ritchie 2007), evolutionary conflicts between the sexes (Parker 1979; Chapman et al. 2003; Parker 2006), kin selection and social interactions (Hughes et al. 2008). In polyandrous mating systems, females mate with multiple males providing the opportunity for both pre- and postcopulatory intra- and intersexual selection, which are reduced or absent under monogamy. Many studies have examined how mating systems influence male and female behavioral, morphological, and physiological traits. Likewise, microarray and sequencing studies have found that genes with higher expression in males, which are usually related to reproduction, show greater rates of both coding sequence and expression divergence among related species compared to unbiased or female-biased genes (Ellegren and Parsch 2007). This pattern has been suggested to be due to the strength of sexual selection on males. However, how mating systems influence female molecular evolution is relatively unknown (Pointer et al. 2013; Hollis et al. 2014). Sex-specific selection arising from mating system variation should also have a strong effect on females and plays a key role in driving the evolution of female reproductive genes (Mank et al. 2013).

Mating system variation can impose divergent sex-specific selection on females in multiple ways. Polyandry commonly increases female fecundity in insects (Arnqvist and Nilsson 2000), though the reasons for this are not well understood. Premating or cryptic female choice can increase the genetic quality, diversity, or compatibility of offspring (Jennions and Petrie 2000; Andersson and Simmons 2006; Slatyer et al. 2011). Thus, polyandry potentially influences both selection for direct and indirect fitness benefits to females, and for female resistance to direct and indirect costs of mating (e.g., Chapman et al. 1995; Crudgington and Siva-Jothy 2000; Wigby and Chapman 2005; Franklin et al. 2012; Lehtonen et al. 2012). Sexually antagonistic effects that arise indirectly from male adaptations to competition can lead to a coevolutionary arms race between the sexes (Holland and Rice 1998). Sexual interactions with multiple males are therefore expected to lead to both strong positive and antagonistic selection on females, which are predicted to influence patterns of gene expression. Despite these predictions, little is known about female evolutionary responses to different mating systems (Kvarnemo and Simmons 2013) and particularly so for gene expression (Mank et al. 2013). While several studies have utilized microarrays to identify genes in females that are involved in sexual interactions, such as those responding to male courtship stimuli (Cummings et al. 2008; Immonen and Ritchie 2012) or mating (McGraw et al. 2004; Mack et al. 2006; McGraw et al. 2008; Innocenti and Morrow 2009; Dalton et al. 2010), the evolution of gene expression due to sexual or sex-specific selection arising from intersexual interactions is poorly understood (Hollis et al. 2014).

Here, we use an experimental evolution approach to manipulate the intensity of selection from sexual interactions above or below the level naturally experienced by females in *Drosophila pseudoobscura* populations for 100 generations, followed by microarray analysis of females, to enumerate and identify the genes and their functions that respond to this experimental manipulation in females. The manipulations were obligate monogamy (M, single male and female housed together) and thus no sexual selection, and elevated polyandry (E; one female housed with six males which is at least twice the number of mates that females have typically been found to mate with in the wild) (2–3 males; Anderson 1974). We expect strong selection in the polyandrous manipulation as a consequence of both intrasexual selection (through both male–male competition and sperm competition) and intersexual selection (through both female choice and cryptic female choice) and sexual conflict. Note that because in each treatment there is only one female, no selection from female–female interactions arises, and thus, any changes in females are likely a direct consequence of intersexual interactions, or due to genetic correlations between the sexes when selection acts on males. Previous work on this system has found divergence in phenotypic traits presumed to be under sexual selection. For example, E males have faster courtship song (Snook et al. 2005), higher courtship frequency (Crudgington et al. 2010), and larger accessory glands (Crudgington et al. 2009) that produce seminal fluid proteins, which in *D. melanogaster* influence male and female fitness (Chapman et al. 1995; Wigby et al. 2009; Avila et al. 2011). Sexual conflict occurs given that E, but not M, males harm M females by reducing the total number of offspring (Crudgington et al. 2010). On the other hand, coevolution with multiple males has benefitted females because E females show higher fecundity and offspring hatching success when mated to ancestral males (i.e., males from a moderately polyandrous mating system), whereas M females do not (Crudgington et al. 2005).

We use this system to examine how selection from mating system variation influences the evolution of the female transcriptome, to estimate how much of the female transcriptome responds to differing selection regimes, and to identify the functions of any responding genes. We use data from a study of both sexes in a wild-type strain of *D. pseudoobscura* to ask if the genes identified as having altered gene expression in females are disproportionally normally female-biased in expression, which is predicted from sex-specific selection but rarely experimentally demonstrated (Hollis et al. 2014).

## Methods

### Mating system treatments

We experimentally manipulated the opportunity for sexual selection and intersexual conflict via changes in the mating system through either enforcing monogamy (1:1 sex ratio with random mate assignment; M) or elevating polyandry (1:6 sex ratio; E) (e.g., Crudgington et al. 2005; Bacigalupe et al. 2008; Crudgington et al. 2009). The design allows for differences in the number of progeny produced by females to be reflected in the composition of the next generation (Crudgington et al. 2005), analogous to natural selection. Potential differences in maternal and environmental effects of experimental flies were controlled by maintaining flies in identical conditions prior to the experiments (Crudgington et al. 2009, 2010). Two of four replicate populations were used in this study for each of the mating system treatments. The number of families contributing to the next generation depends on treatment, an approach that has successfully standardized Ne between treatments (Snook et al. 2009) thus minimizing biases due to drift between treatments. Furthermore, we only conclude responses are due to treatment when they are seen consistently across replicates, and thus, observed evolutionary changes are driven by response to selection rather than to drift.

#### Sample preparation

We used females that had undergone 100 generations of experimental evolution under the M and E selection regimes. Experimental flies were generated using standard densities of 100 first instar larvae per food vial (Crudgington et al. 2010). Virgin flies were collected and sexed under light CO_2_ anesthesia and used for the experiments 5 days after eclosion. The females were anesthetized with CO_2,_ five randomly chosen whole flies per treatment were pooled to form each sample and stored in RNAlater (Qiagen, Düsseldorf, Germany). Three replicate biological samples were prepared for each treatment from each replicate population, resulting in a total of 12 samples. RNA extraction, microarray hybridization, and image scanning were performed by the Liverpool Microarray Facility at the University of Liverpool (see http://www.liv.ac.uk/lmf/protocols.htm for details).

### Analysis

#### Differential expression

We used Agilent 1-color custom 4-plex 44K oligonucleotide microarrays (GEO platform GPL15171) (Jiang and Machado 2009) to test for differential gene expression between the treatments. Microarrays offer the advantage of getting expression information on the gene level using an annotated chip. The array platform contains 45,220 spots with positive and negative controls and oligonucleotide probes representing 18,850 unique gene predictions from the *D. pseudoobscura* genome. Gene annotations were done using the *D. pseudoobscura* genome annotation 2.2 (Jiang and Machado 2009). The array also contains *D. persimilis*-specific probes, which were excluded from our analysis. Both tissue and functional enrichment analyses were done using *D. melanogaster* orthologs (obtained from C. Machado; Jiang and Machado 2009). The microarray data have been submitted to Gene Expression Omnibus with accession number GSE35410.

Packages within Bioconductor (Gentleman et al. 2004) (URL: http://www.bioconductor.org) in R (version 2.13.0) (R Development Core Team 2011) (URL: http://www.R-project.org) were used for data preprocessing and analyses. Raw intensity values were corrected for background hybridization using “normexp” with method = “mle,” and between-array normalization performed using “quantile,” as implemented with package “limma” (Smyth and Speed 2003; Smyth 2005). An average intensity value for annotated replicate probes was calculated with “genefilter” (Gentleman et al. 2011), resulting in a total of 15,734 annotated unique genes to be retained for the analysis.

To test for differential gene expression between the two selection treatments, we fitted a linear model using the “limma” package with empirical Bayes approximation of the standard errors using “eBayes” (Smyth 2004, 2005) and the replicate of the selection regime included as a random factor (Smyth 2005). The *P*-values of the moderated *t*-statistics were adjusted by estimating the false discovery rate (FDR) to control for multiple testing (Benjamini and Hochberg 1995) with a cutoff of <5%.

#### Tissue enrichment

To examine the tissues implicated in differential gene expression response to mating system variation, we used the FlyAtlas dataset (Chintapalli et al. 2007) of *D. melanogaster* to test patterns of tissue enrichment of *D. pseudobscura* orthologs. From the FlyAtlas data, we included only the adult tissues, and a gene was deemed enriched in the focal tissue if it showed at least twofold higher expression relative to whole body (Innocenti et al. 2011). We tested for tissue enrichment among: (1) all the differentially expressed genes, (2) genes upregulated in E females, and (3) genes upregulated in M females. As a further examination of differential tissue-specific responses, we also tested for significant deviance from a 1:1 ratio in the proportion of genes with higher expression in E versus M females for each tissue type, that is, we tested if E and M females are equally likely to upregulate the differentially expressed genes for that tissue type. We used chi-square tests, and all the reported *P*-values are Bonferroni-corrected for multiple testing.

#### Functional enrichment

We performed functional enrichment analyses with Database for Annotation, Visualization and Integrated Discovery (DAVID) (Dennis et al. 2003; Huang et al. 2009) for the datasets of differentially expressed genes. We used the following databases: Gene Ontology (GO) database with levels of “Biological Processes,” “Molecular Functions,” and “Cellular Component”; Integrated Documentation Resource for Protein Families, Domains, Regions and Sites (INTERPO) database; Kyoto Encyclopedia of Genes and Genomes (KEGG), as implemented within DAVID (Dennis et al. 2003; Huang et al. 2009). The overrepresentation of functional annotations among the differentially expressed genes was assessed using the Functional Annotation Clustering tool, which we applied for all the genes (1) upregulated in E or M females and (2) for the tissues overrepresented among the upregulated genes in E or M.

#### Patterns of expression changes in sex-biased genes

To classify genes expressed differentially between M and E females with respect to a wild-type pattern of sex bias, we used a microarray dataset that previously determined the sex bias status of genes in *D. pseudoobscura* (Jiang and Machado 2009), obtained via the SEBIDA database (Gnad and Parsch 2006). This study of sex bias used the same array platform as the present study. Following the original study, we used a false discovery rate (FDR) (Benjamini and Hochberg 1995) cutoff 0.0001% (*q*-value < 0.000001) for identifying sex-biased differentially expressed genes. The sex bias classification was used to test for potential effects of treatment on normally female- and male-biased genes expressed in females. As the direction of sex bias is broadly consistent between closely related species (Jiang and Machado 2009), we consider that data from a study of a wild-type strain of the same species used here will be unlikely to result in any systematic bias in the direction of sex bias gene expression.

We used chi-square tests to determine whether there are disproportionate numbers of sex-biased and unbiased genes among the differentially expressed genes. The expected numbers were calculated based on the proportions of female-, male-, and unbiased genes among the genes included in the analysis of differential expression (15,734), which is close to the number of annotated coding sequences in *D. pseudoobscura* genome (16,071 in annotation 2.2). Chi-square tests were also used to test whether the proportions of female- and male-biased genes upregulated in M versus E females were significantly different from the overall proportions of upregulated genes in each female treatment observed across all differentially expressed genes. In order to test if these patterns are exclusive to genes expressed in the female reproductive system, we repeated the analysis after excluding all the female reproductive tract genes (enriched by at least twofold either in ovaries or in mated/virgin spermatheca) using the FlyAtlas data. Binomial exact tests were used to test whether sex-biased and all of the differentially expressed genes between M and E females show any disproportionate patterns of chromosome distribution. The expected number of genes in each chromosome was calculated based on the pattern observed for chromosome distribution of the genes in the *D. pseudoobscura* genome.

We also compared the expression of the sex-biased genes identified as being differentially expressed between M and E females to wild-type females (female-biased *N* = 996; male-biased *N* = 599), in order to test the putative direction of changes in gene expression as a consequence of mating system variation. The wild-type female gene expression arrays (Jiang and Machado 2009) were normalized together with the experimental female arrays (Rung and Brazma 2013) (following the same method as above), and a linear model was fitted to obtain estimates of expression difference for wild-type females and each of the experimental females using “limma” (Smyth and Speed 2003; Smyth 2005). We categorized the genes of interest based on their sex bias in the wild-type and calculated average expression in each female type for these genes using the normalized intensity values (Fig. 6A–B). We then counted the male- and female-biased genes that showed higher relative expression (logFC) in each of the experimental female types when compared to the wild-type (Fig. 6C) and tested whether the proportion of upregulated female-biased genes differs significantly from upregulated male-biased genes, using chi-square tests.

## Results

**Figure 1:**
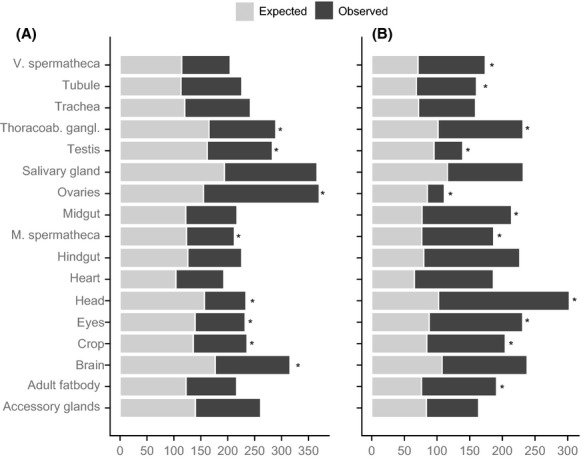
Tissue enrichment among the differentially expressed genes upregulated in (A) polyandrous and (B) monandrous females. Expected values are from chi-square tests, based on *Drosophila pseudoobscura* orthologs in the *D. melanogaster* FlyAtlas dataset. *Bonferroni-corrected *P*-value <0.05.

**Figure A1:**
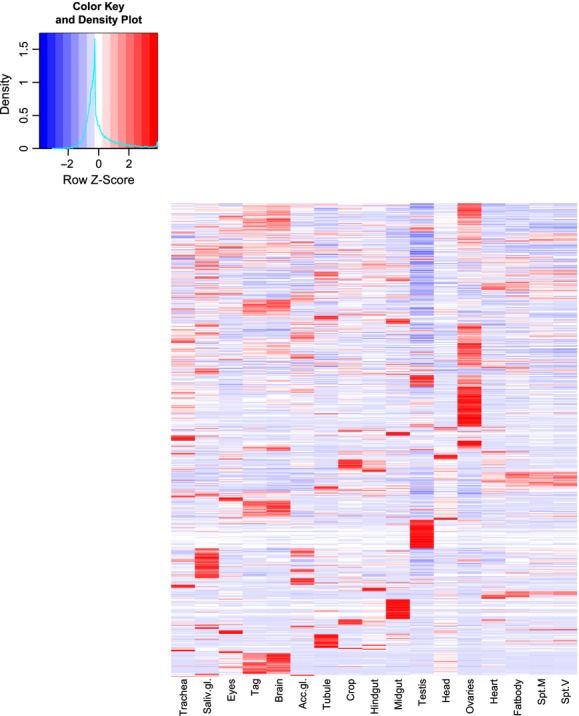
Heatmap of standardized tissue enrichment across all the differentially expressed genes (rows) of the experimental polyandrous and monandrous females. The heatmap shows in which tissue(s) the genes are enriched, highlighting those with the highest numbers of genes, and if the genes are enriched simultaneously in multiple tissues. Increasing red indicates higher enrichment value relative to the whole body (data from FlyAtlas).

We observed large-scale divergence in gene expression between virgin females subjected to enforced monogamy or elevated polyandry for 100 generations: 2280 genes were significantly differentially expressed, which constitutes 14% of the transcriptome (Table S1 for all the differentially expressed genes and Table 1 for the top 50, with annotations on wild-type sex bias and Gene Ontology Biological Process). The differentially expressed genes had a significant overrepresentation of hindgut-, trachea-, and salivary gland-enriched genes and underrepresentation of virgin spermathecal genes, along with the expected underrepresentation of genes associated with male-specific tissues (male accessory gland and testis) (Table A1). Separate analysis by treatment showed that the genes with higher expression in E females contained a significant excess of ovary-enriched genes (Table A2, Fig. 1). Differentially expressed genes enriched in the ovaries were involved in mitosis- and meiosis I-related processes (Table S2). Genes upregulated in M females showed overrepresentation of those expressed in the head, heart, eyes, hindgut, midgut, fatbody, crop, thoracic ganglion, trachea, and virgin spermatheca (Table A2, Fig. 1). The functional terms associated with the differentially expressed genes enriched in these tissues are presented in Tables S3-12. Some of these tissues show correlated expression profiles (Figs. A1, 8 A2) and have many functional terms in common. For example, the head- and eye-enriched genes shared terms associated with visual perception (Tables S3-4), and the hind- and midgut genes with metabolic processes (Tables S5-6). Consistent with these patterns, similar functional terms were significantly enriched when all the genes upregulated in E (Table S13) or M (Table S14) females were considered separately without *a priori* classification of genes by tissue. Overall, these results suggest that selection due to elevated polyandry has favored higher expression in E females of genes that are associated with ovaries and ovarian function while selection under monandry has favored higher expression of genes in females associated with somatic tissues.

**Table 1:** Fifty most significantly differentially expressed genes between experimental polyandrous (E) and monandrous (M) females (FDR adjusted *p* value <0.000003)

GA number	logFC(E/M)	GO BP	Wt sex bias
GA16027	2.30		M
GA14428	1.87		Ub
GA18222	1.70		F
GA19985	1.46		F
GA22618	1.34		Ub
GA11784	1.25	Defense to fungus (m)	F
GA12421	1.18	Actin cytoskeleton organization (m)	F
GA13248	1.08	Sleep (m)	F
GA15295	1.06		Ub
GA23473	0.90		Ub
GA17206	0.89	Wound healing (m)	F
GA27664	0.89		Ub
GA12788	0.77		Ub
GA23837	0.76	Neurogenesis (m)	F
GA20067	0.75		Ub
GA20664	0.69	Notch signaling (gi), biological regulation (m)	Ub
GA13111	0.68	Regulation of transcription (ss)	M
GA22876	0.64		Ub
GA14417	0.61	Mitosis (m)	F
GA22002	0.58	Lipid metabolic process (ea)	F
GA11182	0.53	Nucleic acid binding (ea)	F
GA11060	0.52	Adult life span (m), locomotor behavior (m)	M
GA18032	0.52	DNA damage repair (m)	F
GA20641	0.52	Apoptosis (m)	Ub
GA22300	0.51	Actin filament organization (m), behavioral response to ethanol and nicotine (m)	F
GA20233	0.48	Oxidation–reduction (ea)	F
GA11554	0.47		F
GA16655	0.45	Oxidation–reduction (ea)	F
GA12981	0.44		F
GA14866	0.44		M
GA13074	0.43	Mitosis (m), lateral inhibition (m), neurogenesis (m)	F
GA13258	0.42	Regulation of transcription (m)	F
GA27130	0.39		F
GA16770	0.30		F
GA17499	−0.42	Brain development (m), associative learning (m), long-term memory (m)	F
GA20879	−0.50		F
GA13844	−0.54	Positive regulation of Notch signaling pathway (m)	F
GA17094	−0.54	Neurogenesis (m)	F
GA24337	−0.63	Alternative splicing (m)	Ub
GA18033	−0.72	Sleep (m), locomotor behavior (m)	M
GA24833	−0.91		M
GA11415	−1.00	Flight behavior (m), visual perception (m), cuticle pigmentation (m), dopamine biosynthetic process (m)	Ub
GA24793	−1.02		M
GA18512	−1.21	Phagocytosis (m), retinal metabolic process (m)	M
GA27173	−1.56		M
GA19482	−1.78	Neurogenesis (ea)	M
GA26669	−2.23	Lipid catabolic process (m)	M
GA25502	−2.43	Circadian rhythm (m, da, gi), mitosis (m), regulation of lipid metabolism (m)	Ub
GA29241	−2.95	Gravitaxis (m)	Ub
GA25504	−3.34		Ub

Ub, unbiased; F, female-biased; M, male-biased.

logFC = log2 of fold difference in expression with positive values indicating higher relative expression in E and negative in M females. GO BP = representative Gene Ontology term for Biological Process (from FlyBase using *D. melanogaster* ortholog annotation); evidence inferred from (m) mutation phenotype, (gi) genetic interaction, (da) direct assay, (ea) electronic annotation, (ss) structural similarity. Wt sex bias from Jiang and Machado (2009).

**Figure 2:**
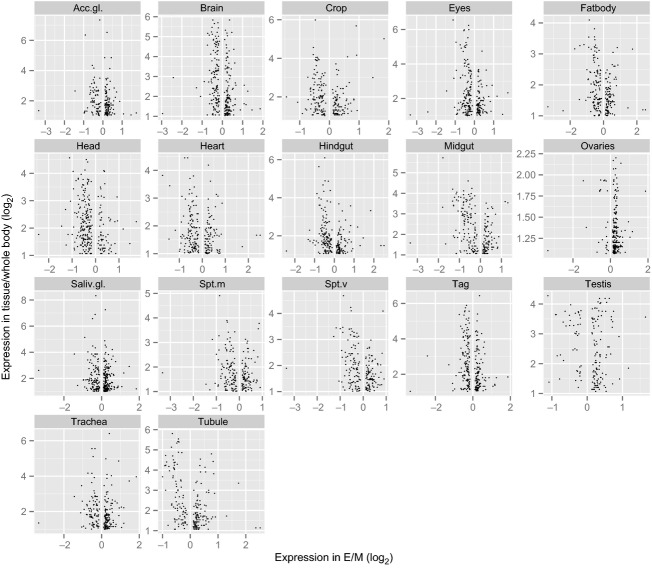
Differential expression of genes between polyandrous (E) and monandrous (M) females by tissue type (tissue-specific expression data from FlyAtlas).

**Figure A2:**
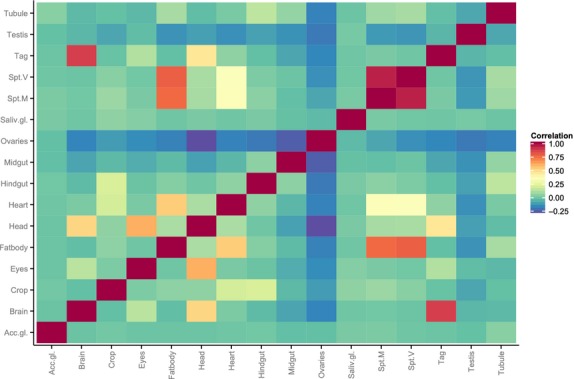
Correlation matrix of tissue expression enrichment for all the differentially expressed genes of E and M, indicating the extent of correlation between tissues for these genes.

We also tested for deviance from 1:1 in the proportion of differentially expressed genes upregulated in each female type for each tissue. Consistent with the above tissue enrichment analysis, E females had a significantly higher proportion of upregulated genes in the ovaries but also in the salivary gland and trachea, whereas M females showed a significantly higher proportion of upregulated genes in the eyes, head, heart, hindgut, and midgut (Table A2, Fig. 2). While genes normally expressed in male-specific tissues are, as expected, underrepresented among the differentially expressed genes of females overall (Table A1), we observe a few hundred such genes (e.g., testis and accessory glands) with higher expression in E females (Fig. 2). These male tissue-specific differentially expressed genes are either uncorrelated or negatively correlated with expression levels in other tissues (Figs. A1, A2), and therefore, it is unknown in which tissues these genes might be expressed in *D. pseudoobscura* females.

Changes in levels of tissue-specific gene expression could reflect changes in gene regulation, copy number of relevant genes, or the relative amount of tissue. One possible explanation for our findings is differences in tissue allocation, for example, if E females have more ovary tissue material than M females as a response to evolution under polyandrous conditions. To examine this, we counted the numbers of ovarioles in available samples of females from each treatment from the same generation as the microarray samples. There was no significant difference in ovariole number between the selection treatments (fitted means from a GLM = 38.89 for E females, 36.90 for M, pooled s.e. 1.70; *F*_1,68_ = 1.38; *P* = 0.245). Previous work has demonstrated no difference between treatments in the size of female sperm storage organs (Crudgington et al. 2009; Snook et al. 2010). However, we did find a significantly higher number of eggs per ovariole in E than in M females (fitted means from GLM = 1.50 for E and 0.89 for M, pooled SE = 0.23; *F*_1, 44_ = 9.26; *P* = 0.0039).

**Figure 3:**
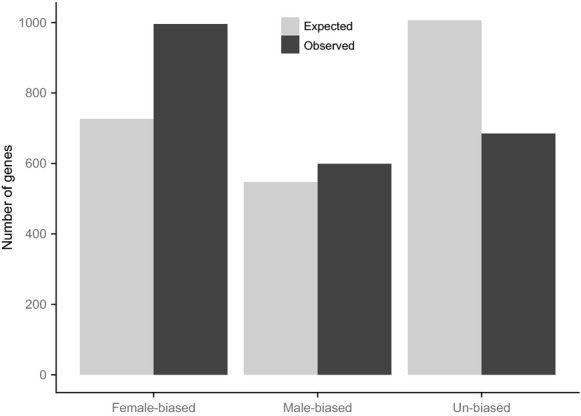
The numbers of observed and expected sex- and unbiased genes among the differentially expressed genes between the polyandrous and monandrous females (*χ*^2^_(2)_ = 207.6, *P* < 0.0001). Expected numbers are based on the patterns observed for *D. pseudoobscura* in Jiang and Machado (2009).

**Figure 4:**
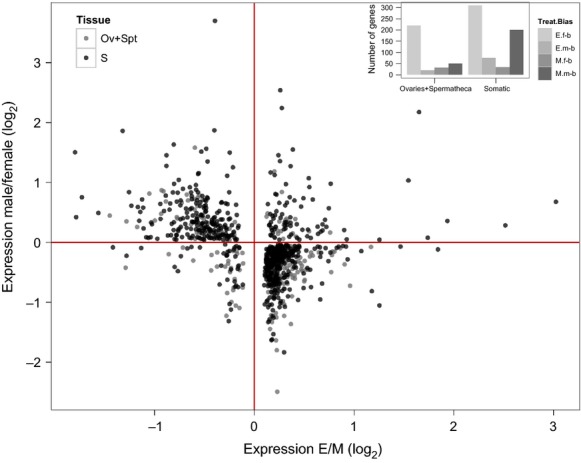
Main figure: Gene expression difference between polyandrous (E) and monandrous (M) females by sex bias, separately for somatic (S) and ovary- + spermathecae-enriched genes (Ov + Spt). Insert: the numbers of differentially expressed genes upregulated in E and M treatment (Treat) by sex bias status (E.f-b = E female-biased; E.m-b = E male-biased; M.f-b = M female-biased; M.m-b = M male-biased) separately for somatic and female reproductive tissues. The figures show how the relative difference in the upregulation of female- and male-biased genes is seen for both female reproductive tract- and somatic-tissue-enriched genes. Tissue data from FlyAtlas (Chintapalli et al. 2007).

Using a previous dataset describing sex-biased gene expression in this species (Jiang and Machado 2009), we found disproportionate numbers of normally sex-biased genes among the differentially expressed genes in females: 1595 genes (70% of those that showed differential expression) were sex-biased with a 37% excess of normally female-biased, a 9% excess of normally male-biased genes, and a 32% deficit of normally unbiased genes (*χ*^2^_(2)_ = 207.6, *P* < 0.0001, Fig. 3). We further analyzed this to ask whether sexual selection treatment resulted in differences in either the number of differentially expressed genes or the relative magnitude of expression change, for each gene category. E and M females showed opposing patterns of expression changes among these sex-biased genes in terms of gene numbers; more normally, female-biased genes were upregulated in E compared to M females (*χ*^2^_(1)_ = 290.7, *P* < 0.0001, total gene number for E = 872; for M = 124), whereas more normally male-biased genes were upregulated in M compared to E females (*χ*^2^_(1)_ = 280.6, *P* < 0.0001, total gene number for M = 432; for E = 167). These patterns are not only caused by genes enriched in the female reproductive tissues; they are at least as pronounced among normally sex-biased somatic genes (*χ*^2^_(1)_ = 119.1, *P* < 0.0001; male-biased: *χ*^2^_(1)_ = 131.7, *P* < 0.0001, Fig. 4; gene numbers are counted from ortholog data using FlyAtlas, Chintapalli et al. 2007). Thus, these results suggest that more of both female reproductive tract and somatic tissue female-biased genes are upregulated in the experimental polyandrous females. Monandrous M females on the other hand have evolved higher expression in a greater number of normally male-biased genes (Fig. 4), enriched in a variety of somatic tissues including the head, gut, and fatbody (see above, Table A2).

Interestingly, this overall pattern (M females upregulating relatively more male-biased genes and E females upregulating female-biased genes) does not hold for the few hundred male-specific tissue-enriched genes (testis and accessory glands). These genes are significantly overrepresented among the normally male-biased genes upregulated in E (*χ*^2^_(1)_ = 4.4, *P* = 0.03) but underrepresented among those upregulated in M females (*χ*^2^_(1)_ = 9.3, *P* = 0.002), relative to somatic male-biased genes (Table A2).

**Figure 5:**
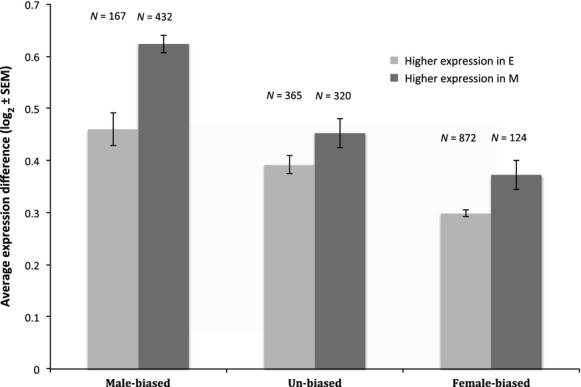
The average expression magnitude difference (with ±SE) between M and E females by sex bias (as defined in the wild-type), separately for the genes that show higher expression in E and in M.

In addition to the number of genes, we also tested whether the magnitude of differences in gene expression changes in each gene category differed between the treatments. For each differentially expressed gene, we categorized which treatment upregulated that gene. We then categorized whether these genes were normally male-biased, female-biased, or unbiased and averaged the logfold expression difference of the treatments across genes for each category. We found that, regardless of sexual selection treatment, normally male-biased genes had a greater average *difference* between treatments compared to both female- and unbiased genes (Fig. 5). However, we also found that genes with higher expression in M females exhibit greater average expression differences relative to E females across all gene categories, and particularly for normally male-biased genes (Fig. 5). The genes enriched in male-specific tissue are no exception (result not shown). Thus mating system variation results in both the number of differentially expressed genes and the magnitude of such change, although this depends on sexual selection treatment; E females have a greater number of female-biased genes that are upregulated compared with M females, whereas M females have a greater magnitude of change in those female-biased genes that they upregulate.

**Figure 6:**
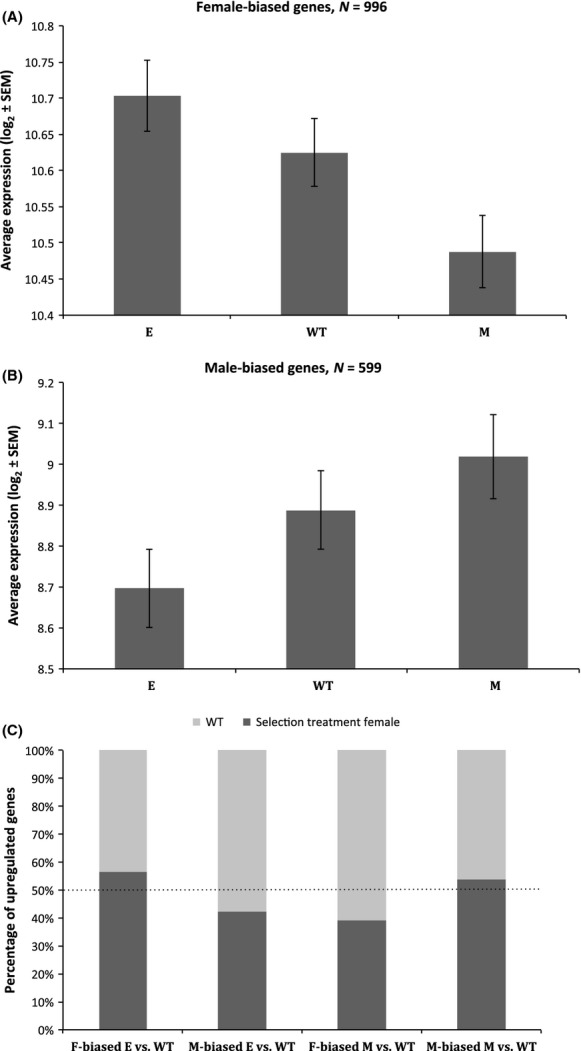
Expression patterns between experimental and wild-type females for the differentially expressed genes of E and M that have a sex-biased status in the wild-type. Average expression in each female type for the female-biased genes (A) and male-biased genes (B). Percentage of the female-biased (F) and male-biased (M) genes upregulated in each of the selection treatment female groups relative to the wild-type (C).

To determine the putative direction of evolutionary change in the naturally sex-biased gene expression in females due to mating system manipulation, we compared the expression levels of E and M females to those of the wild-type females (from the original study of sex bias, Jiang and Machado 2009) who have natural levels of polyandry. The level of expression of the 996 female-biased and 599 male-biased differentially expressed genes is changed in opposite directions for E and M, such that the wild-type (WT) expression pattern is intermediate to both mating system manipulations (Fig. 6A–B). Hence, the experimental female types differently upregulate the sex-biased genes relative to WT: E females upregulate a significantly greater proportion of the normally female-biased genes than of the normally male-biased genes (*χ*^2^_(1)_ = 29.3, *P* < 0.0001, Fig. 6C). The reverse is true for M females who upregulate a significantly lower proportion of the normally female-biased genes than of the normally male-biased genes relative to WT (*χ*^2^_(1)_ = 31.7, *P* < 0.0001, Fig. 6C). Thus, these results indicate that both E and M females have diverged away from the wild-type pattern. Many methodological differences (e.g., age of flies, temperature, RNA processing) as well as population divergence can result into differences in gene expression between our experimental females and wild-type females of Jiang and Machado (2009). However, such factors are unlikely to confound our interpretation, because our experimental females differ from the wild-type to *opposite* directions for the genes tested, rather than to the same, which would be predicted by methodological or population differences.

Theory predicts that female-beneficial alleles should accumulate on the X chromosome (in an XY system), because X-linked loci spend two thirds of their time in females (Rice 1984). In accordance with this, studies of *Drosophila,* including *D. pseudoobscura* (Jiang and Machado 2009), have found enrichment of female-biased genes on the X (Ellegren and Parsch 2007; Meisel et al. 2012). We tested whether female-biased genes in females that respond to mating system variation predominantly reside on the X, but found no support for this on either the ancestral XL arm or the neo sex chromosome XR (Exact binomial test: XL: *P* = 0.53, *N* = 177; XR: *P* = 0.67, *N* = 192). Likewise, differentially expressed female-biased genes did not show any significant disproportionate patterns on autosomes (2nd: *P* = 0.09, *N* = 252; 3rd: *P* = 0.90, *N* = 177; 4th: *P* = 0.21, *N* = 154). For male-biased genes, we found a deficit on the 4th chromosome but no other significant patterns (XL: *P* = 0.52, *N* = 79, XR: *P* = 0.51, *N* = 89, 2nd = 0.10, *N* = 166; 3rd: *P* = 0.53, *N* = 119; 4th: *P* = 0.04, *N* = 116). For unbiased differentially expressed genes, there was a significant deficit of genes on the neo sex chromosome XR but no other deviations (XL: *P* = 0.13, *N* = 101, XR: *P* = 0.01, *N* = 71, 2nd = 0.07, *N* = 86; 3rd: *P* = 0.11, *N* = 91; 4th: *P* = 0.54, *N* = 72).

## Discussion

We compared the gene expression between experimentally evolved monandrous and polyandrous virgin females to test how selection from mating system variation affects the female transcriptome. Our results demonstrate that evolution of the female transcriptome occurs rapidly, with 14% of the transcriptome changing in expression pattern after only 100 generations of selection. Comparison with a wild-type population suggests that these changes occur in both monandrous and polyandrous females.

In order to characterize the genes involved in response to mating system variation, we used information on tissue (Chintapalli et al. 2007) and functional (Chintapalli et al. 2007; Huang et al. 2009) enrichment from *D. melanogaster* and sex-biased gene expression in wild-type *D. pseudoobscura* (Jiang and Machado 2009). Genes likely to be normally female-biased are over 30% more common than expected among the differentially expressed genes, and majority of these are upregulated in polyandrous females relative to monandrous. In contrast, genes upregulated in monandrous females relative to polyandrous are more likely to be male-biased. A striking exception to this pattern is that, while genes enriched in male-specific tissues are underrepresented overall among the differentially expressed genes, such genes show higher relative expression in polyandrous females. Hence, we have demonstrated using experimental evolution that targets of altered sexual selection regimes in females disproportionately include genes that are normally sex-biased in expression.

In addition to the total number of genes that were differentially expressed between treatments, we also examined the magnitude of change in these genes that were categorized as normally male-, female-, or unbiased. We found that monandry generally results in a larger magnitude of upregulation for all differentially expressed gene categories. Thus, monandry has selected for both greater numbers of normally male-biased genes being more highly expressed relative to polyandrous females but also resulted in a greater magnitude of change across all responsive genes. In contrast, polyandry has selected for higher expression in more genes that are normally female-biased with the exception of genes that are normally enriched in male reproductive tissues, where most of these were upregulated in polyandrous females. The function of such normally male-biased genes in females is clearly of great interest. One explanation may be that they are expressed indirectly due to greater sexual selection on polyandrous males, and thus, the changes we observe in females may reflect not only female-specific evolutionary responses but also potentially divergent intersexual genetic correlations.

We find that ovary-enriched genes are overrepresented among the genes with higher expression in polyandrous females, whereas many adult somatic tissues, including head, fatbody, and gut, are overrepresented among the genes upregulated in monandrous females. These patterns suggest that changing the strength of sexual and sex-specific selection may have changed the relative investment into the reproductive and somatic tissues in females or processes regulated in these tissues. In support of this, we find that polyandrous virgin females have a higher number of eggs per ovariole than monandrous females (but no difference in the number of ovarioles). Previous work has shown that polyandrous E females have higher fecundity than M when mated to nonexperimental males (Crudgington et al. 2005). Our finding suggests these differences likely arise from initial difference in the investment to egg production and not (only) in response to mating. Differential expression of mitosis- and meiosis I-related genes in the ovaries reflects this. Some of the genes are also likely involved in maternal provisioning of mRNAs and proteins into the developing eggs (Preuss et al. 2012). Genes involved in transcription of mRNAs transferred into unfertilized eggs are often female-biased but intriguingly can also involve some commonly associated with male reproduction (Preuss et al. 2012), which can offer another explanation for our finding that polyandrous females upregulate some male tissue-enriched genes.

Sex-specific gene regulation is thought to “resolve” intralocus sexual conflict (Ellegren and Parsch 2007), which we would expect to be greater in the polyandrous lines. Overall, we found more normally female-biased genes and fewer normally male-biased genes, upregulated in experimental polyandrous females, consistent with this hypothesis. Wild-type females appear intermediate in expression between the E and M females, likely because E and M females have evolved in opposite directions from each other. Studies of gene expression patterns in males of these lines are necessary to assess fully the patterns of sexual dimorphism in the transcriptome. Hollis et al. (2014) found reduced sexually dimorphic gene expression in polyandrous *D. melanogaster* males and females compared to experimental monogamy individuals and concluded that monandry allows females to evolve closer to an optimum (more feminized) gene expression pattern when intralocus sexual conflict is reduced. Our finding of higher expression of female-biased and lower of male-biased genes in E females contrasts with this. Both species experience interlocus sexual conflict under polyandrous conditions (Holland and Rice 1998, 1999; Crudgington et al. 2005, 2010), but they differ in other consequences of polyandry. For example, female *D. pseudoobscrua* (Gowaty et al. 2010), but not *D*. *melanogaster* (Brown et al. 2004), benefit from multiple mating. They may also differ in the extent to which the sexes experience genetic correlations in sex-biased gene expression and thus how “free” the sexes are to evolve independently. We encourage more work to identify the fitness consequences of evolution under different mating systems for females and the mechanisms mediating these, before general conclusions about how females respond to variation in mating systems can be made.

## Summary

Here, we show that selection from sexual interactions with males shapes the female transcriptome, with virgin female gene expression changing rapidly under altered mating systems. Some of the expression changes could be affected by genetic correlations between the sexes, because we find higher expression for genes enriched in male reproductive tissues in polyandrous females. However, most of the patterns we observe are more compatible with sex-specific selection acting on females. The expression changes are more numerous for female-biased genes with a strong signal from the ovaries. Monandrous females show reduced expression in the ovaries and processes related to mitosis and meiosis and higher expression in the head and gut associated with visual perception and metabolism, respectively. These patterns suggest changes in the relative investment between reproductive and somatic processes, and in support for this, we find that polyandrous virgin females have higher relative number of eggs in the ovaries compared to monandrous (but no differences in the amount of ovary tissue). Previous studies comparing the patterns of molecular evolution of sex-biased genes between species have suggested faster evolution of male-biased genes due to sex-specific selection on males (Ellegren and Parsch 2007; but see Mank et al. 2007; Zhang et al. 2007). However, sex-specific selection arising from mating system variation should also have a strong effect on females and play a fundamental role in driving different patterns of genome expression between the sexes (Mank et al. 2013). The rapidity and extent of the transcriptome changes observed in this study show that “fast female” evolution can also occur.

## Appendix

**Table A1:** Tissue enrichment patterns among all the differentially expressed genes of polyandrous and monandrous females

Tissue	Chisq	Obs	Exp	Bonf p-val	Enrichment
Accessory glands	18.9	201	260	0.0002	UNDER
Adult fatbody	0.2	209	202	n.s.	
Brain	0.2	269	262	n.s.	
Crop	0.006	220	221	n.s.	
Eye	6.6	236	204	n.s.	
Head	0.3	277	286	n.s.	
Heart	1.5	210	226	n.s.	
Hindgut	8.7	247	210	0.053	OVER
Spermatheca mated	0.8	199	188	n.s.	
Midgut	5.5	232	203	n.s.	
Ovaries	2.1	241	261	n.s.	
Salivary gland	10.7	288	244	0.02	OVER
Thoracic ganglion	0.5	254	245	n.s.	
Testis	17.2	165	218	0.0006	UNDER
Tubule	3.2	205	183	n.s.	
Trachea	9.8	209	173	0.03	OVER
Spermatheca virgin	52.6	193	297	7.1E-12	UNDER

**Table A2:** Tissue enrichment of the differentially expressed (DE) genes among polyandrous (*E*) and monandrous (*M*) females. The expected numbers are based on the numbers of genes enriched in each tissue in the FlyAtlas dataset. *Deviation from 1:1*: tests for differences in the relative proportion of genes up-regulated in polyandrous and monandrous females out of all DE genes enriched in that tissue. For example, there are 165 differentially expressed genes that are enriched in the testis, of which 121 are up-regulated in E and 44 in M. This test compares whether their respective proportions out of the total number of 165 genes differ from one another

TEST	DE genes with higher expression in E				DE genes with higher expression in M				Deviation from 1:1	
			
Tissue	Obs.	Exp.	*χ*^2^	Bonf *P*-value	Obs.	Exp.	*χ*^2^	Bonf *P*-value	*χ*^2^	Bonf *P*-value
Accessory glands	**121**	140	3.2	n.s.	80	84	0.15	n.s.	15.9	0.001
Adult fatbody	94	122	8.2	0.07	115^1^	76	24.0	1.62E-05	3.8	n.s.
Brain	139	177	10.8	0.02	130	108	5.8	n.s.	0.5	n.s.
Crop	100	136	12.0	0.009	120^1^	84	18.6	0.0002	3.3	n.s.
Eyes	93	139	19.7	0.0002	**143**^1^	88	43.1	8.80E-10	20.3	0.0001
Head	77	157	53.6	4.08E-12	**200**^1^	102	121.8	4.39E-27	107.5	5.98E-24
Heart	89	104	2.4	n.s.	**121**^1^	65	56.7	8.63E-13	9.2	0.04
Hindgut	100	126	6.6	n.s.	**147**^1^	80	70.4	8.24E-16	17.1	0.0006
Spermatheca mated	89	123	11.8	0.01	110	77	17.5	0.0005	4.0	n.s.
Midgut	95	122	7.4	n.s.	**137**^1^	77	57.2	6.70E-13	14.5	0.002
Ovaries	**215**^1^	155	30.5	5.60E-07	26	85	51.3	1.36E-11	293.3	1.61E-64
Salivary gland	**172**	194	3.3	n.s.	116	116	0.002	n.s.	21.0	7.78E-05
Thoracic ganglion	124	165	13.7	0.004	130^1^	102	10.1	0.02	0.2	n.s.
Testis	**121**	162	13.7	0.004	44	95	35.2	5.09E-08	70.0	1.00E-15
Tubule	113	113	0.00007	n.s.	92	72	9.6	n.s.	3.9	n.s
Trachea	**122**	120	0.02	n.s.	87^1^	68	3.7	0.03	11.1	0.01
Spermatheca virgin	90	115	6.4	n.s.	103^1^	71	17.4	0.0005	1.5	n.s.

1 Indicates overrepresentation in tests for E and M.

Bold text highlights the female selection type that shows significantly higher relative proportion of up-regulated genes in the focal tissue.

## Supporting Information

**Table S1.** All the differentially expressed genes between experimentally evolved polyandrous (E) and monandrous (M) females of *D. pseudoobscura*. **Table S2–12**. Clusters of enriched functional annotation terms (DAVID) for all the differentially expressed genes by tissue. The tissues presented are those identified as significantly overrepresented among the upregulated genes either in E or in M relative to the other female (Table A2, marked with*). **Table S13**. Clusters of enriched functional annotation terms (DAVID) for all the differentially expressed genes upregulated in E females relative to M females. **Table S14**. Clusters of enriched functional annotation terms (DAVID) for all the differentially expressed genes upregulated in M females relative to E females.
