# Supplementary material for: Mating system variation drives rapid evolution of the female transcriptome in Drosophila pseudoobscura
Source: Ecol Evol. 2014 May 6;4(11):2186–201. doi: 10.1002/ece3.1098 (PMC4201433; doi:10.1002/ece3.1098)
Supplement: Supplementary file 2 [file ece30004-2186-sd2.docx]

**Mating system variation drives rapid evolution of the female transcriptome in *Drosophila pseudoobscura***

Immonen, E., Snook, R. R., Ritchie, M. G.

**Legends for Supplementary Tables**

(file: ‘Tables_S1-S14_Immoenetal.xlsx’)

**Table S1.** All the differentially expressed genes between experimentally evolved polyandrous (E) and monandrous (M) females of *D. pseudoobscura*.

**Table S2-12**. Clusters of enriched functional annotation terms (DAVID) for all the differentially expressed genes by tissue. The tissues presented are those identified as significantly overrepresented among the up-regulated genes either in E or in M relative to the other female (Table A2, marked with*).

**Table S13**. Clusters of enriched functional annotation terms (DAVID) for all the differentially expressed genes up-regulated in E females relative to M females.

**Table S14**. Clusters of enriched functional annotation terms (DAVID) for all the differentially expressed genes up-regulated in M females relative to E females.
